# DAG/PKCδ and IP3/Ca^2+^/CaMK IIβ Operate in Parallel to Each Other in PLCγ1-Driven Cell Proliferation and Migration of Human Gastric Adenocarcinoma Cells, through Akt/mTOR/S6 Pathway

**DOI:** 10.3390/ijms161226116

**Published:** 2015-12-01

**Authors:** Lianzhi Dai, Luhua Zhuang, Bingchang Zhang, Fen Wang, Xiaolei Chen, Chun Xia, Bing Zhang

**Affiliations:** 1 Medical School, Xiamen University, Fujian 361102, China; 24520131153431@stu.xmu.edu.cn (L.D.); zhuangluhua@gmail.com (L.Z.); bczhang@foxmail.com (B.Z.); 24520141153450@stu.xmu.edu.cn (F.W.); 2 Zhongshan Hospital, Xiamen University, Fujian 361004, China; cxl910904@gmail.com

**Keywords:** PLCγ1, DAG/PKCδ, IP3/Ca^2+^/CaMK IIβ, Akt/mTOR/S6, cell proliferation, migration, human gastric adenocarcinoma cells

## Abstract

Phosphoinositide specific phospholipase Cγ (PLCγ) activates diacylglycerol (DAG)/protein kinase C (PKC) and inositol 1,4,5-trisphosphate (IP3)/Ca^2+^/calmodulin-dependent protein kinase II (CaMK II) axes to regulate import events in some cancer cells, including gastric adenocarcinoma cells. However, whether DAG/PKCδ and IP3/Ca^2+^/CaMK IIβ axes are simultaneously involved in PLCγ1-driven cell proliferation and migration of human gastric adenocarcinoma cells and the underlying mechanism are not elucidated. Here, we investigated the role of DAG/PKCδ or CaMK IIβ in PLCγ1-driven cell proliferation and migration of human gastric adenocarcinoma cells, using the BGC-823 cell line. The results indicated that the inhibition of PKCδ and CaMK IIβ could block cell proliferation and migration of BGC-823 cells as well as the effect of inhibiting PLCγ1, including the decrease of cell viability, the increase of apoptotic index, the down-regulation of matrix metalloproteinase (MMP) 9 expression level, and the decrease of cell migration rate. Both DAG/PKCδ and CaMK IIβ triggered protein kinase B (Akt)/mammalian target of rapamycin (mTOR)/S6 pathway to regulate protein synthesis. The data indicate that DAG/PKCδ and IP3/Ca^2+^/CaMK IIβ operate in parallel to each other in PLCγ1-driven cell proliferation and migration of human gastric adenocarcinoma cells through Akt/mTOR/S6 pathway, with important implication for validating PLCγ1 as a molecular biomarker in early gastric cancer diagnosis and disease surveillance.

## 1. Introduction

Human gastric cancer is the second leading cause of cancer death and the fourth most prevalent malignancy worldwide [1]. Many factors including the pathogenesis of gastric cancer, diagnosis, and treatment approaches result in the high incidence and mortality rates of gastric cancer [2,3]. Recent literature has shown the involvement of important signal molecules in the pathogenesis of gastric cancers, which is beneficial to developing efficacious molecular biomarkers for early gastric cancer diagnosis and disease surveillance. As an example, the expression of cyclin D1, p21 and p27, alone or in combination, are early events in gastric tumorigenesis and may serve as a candidate molecular marker for the early gastric carcinoma [4]. Mitogen-activated protein kinase (MAPK) kinase 4 (MKK4) kinase expression could serve as a significant prognostic factor for disease-free survival and for overall survival in human gastric cancer [5]. However, the expressions of these molecules are not always in accordance with the pathological progression of gastric cancers. For example, the expression of cyclin D1, p21 and p27 inversely correlated with the lymph node metastasis [4], to the extent that the application of molecular biomarkers could be decreased, due to their complex regulatory mechanism. Hence, studying the expressions of important signal molecules in the pathogenesis of gastric cancer and understanding the underlying transduction mechanism are required to validate the molecular biomarkers.

Phosphoinositide specific phospholipase Cγ (PLCγ), one of PLCs family, has two isoforms, PLCγ1 and PLCγ2. PLCγ1 is ubiquitously expressed in mammalian cells, and has been reported to be highly expressed in some tumor tissues, including colorectal cancer, squamous cell carcinoma, and breast cancer, regulating cancer cell metabolism [6,7,8]. As an example, elevated content of PLCγ1 in colorectal cancer tissues is observed [6]. PLCγ1 is required for the epidermal growth factor receptor (EGFR)-induced squamous cell carcinoma cell mitogenesis [7]. PLCγ mediates high levels of glucose and insulin-induced cell proliferation and migration in MDA-MB-468 breast cancer and SW480 colon cancer cells *in vitro* [8]. Our previous study also showed the higher expression of PLCγ1 in human gastric adenocarcinoma tissue and that the metastasis of human gastric adenocarcinoma cells partly depends on PLCγ1 expression [9]. Moreover, it has been shown that the depletion of PLCγ expression or inhibition of its activity not only significantly increases cisplatin-induced apoptosis but also suppresses the invasive ability of RhoGDI2-overexpressing SNU-484 gastric cancer cells [10]. Therefore, PLCγ may be a potential molecular biomarker in human gastric cancer, and understanding its regulatory mechanism is beneficial to confirm its implication in early cancer diagnosis and monitoring.

PLCγ is activated by many growth factor receptors, including epidermal growth factor (EGF), platelet derived growth factor (PDGF), nerve growth factor (NGF), and type I insulin-like growth factor (IGF-1), and induces hydrolysis of phosphatidylinositol 4,5-bisphosphate (PtdIns(4,5)P2) to form the second messengers diacylglycerol (DAG) and inositol 1,4,5-trisphosphate (IP3), which in turn activate protein kinase C (PKC) and intracellular calcium mobilization, respectively [11,12,13,14,15,16]. Activated DAG/PKC and IP3/Ca^2+^/CaMK II axes, the two classical axes of PLCγ, regulate important events of cancer cell metabolism [17,18]. As an example, activated PLCγ by interleukin-8 generates DAG and IP3, which in turn trigger PKC and the release of calcium from the endoplasmatic reticulum, respectively, and participates in human T24 bladder carcinoma cell migration [17]. In estrogen receptor α (ERα)-positive (ERα(+)) cancer cells, 3,3-*bis*(4-hydroxyphenyl)-7-methyl-1,3-dihydro-2*H*-indol-2-one (BHPI) rapidly hyperactivates plasma membrane PLCγ, generating IP3, which opens EnR IP3R calcium channels, rapidly depleting EnR Ca^2+^ stores [18]. However, the underlying mechanism of DAG/PKCδ and IP3/Ca^2+^/CaMK IIβ axes in PLCγ-driven cell proliferation and migration of human gastric adenocarcinoma cells has not been elucidated.

In this study, we examined the role of PKCδ and CaMK IIβ signal molecules in cell proliferation and migration of human gastric adenocarcinoma cells, using the BGC-823 cell line. Furthermore, the regulatory mechanism related to Akt, mTOR, and S6 signal molecules was investigated. Consequently, both DAG/PKCδ and IP3/Ca^2+^/CaMK IIβ operate in parallel to each other in PLCγ1-driven cell proliferation and migration of human gastric adenocarcinoma cells.

## 2. Results

The effect of inhibiting PKCδ and CaMK IIβ on PLCγ1-driven cell proliferation and apoptosis in human gastric adenocarcinoma cells.

**Figure 1:**
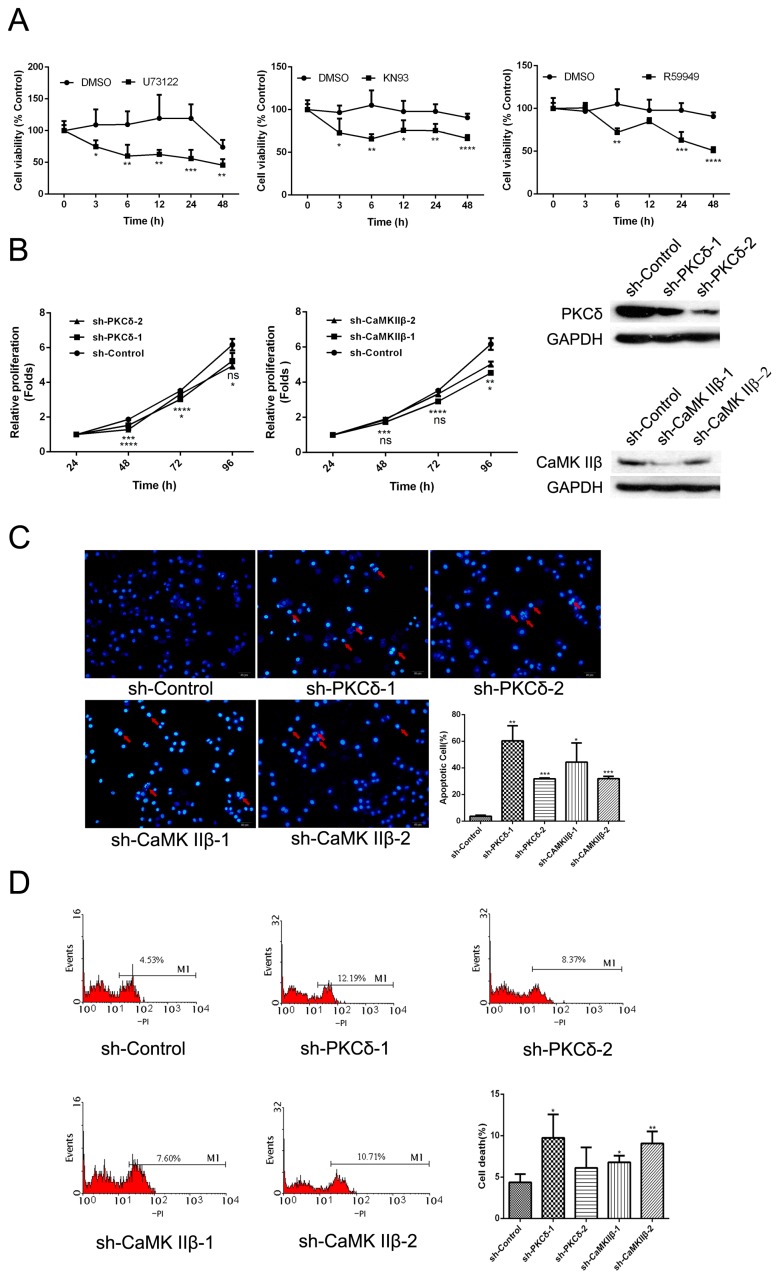
The effect of inhibiting CaMK IIβ and DAG/PKCδ on cell proliferation and apoptosis in human gastric adenocarcinoma. (**A**) Cells were exposed to DMSO (2 μL), U73122 (10 μM), KN93 (16 μM), or R59949 (10 μM) for different time points, respectively. Cell viability was then measured by an MTT assay as described in Materials and Methods; (**B**) Cells were transfected with sh-PKCδ or sh-CaMK IIβ vectors for different time points. Cell viability was measured using an MTT assay as described in Materials and Methods; (**C**) Cells were transfected with sh-PKCδ or sh-CaMK IIβ vectors for 48 h, followed by DAPI staining and counting under OLYMPUS 41 microscope as described in Materials and Methods. The cell nuclei were stained by DAPI staining (blue), and the apoptotic bodies were indicated by red arrows (magnification 200×); (**D**) Cells were transfected with sh-PKCδ or sh-CaMK IIβ vectors for 48 h, followed by PI staining. The cell apoptosis index was analyzed by flow cytometry as described in Materials and Methods. Data are expressed as mean ± S.D. of three independent experiments, each yielding similar results (* *p* < 0.05, ** *p* < 0.01, *** *p* < 0.001, **** *p* < 0.0001, *versus* control).

To investigate whether DAG/PKCδ and IP3/Ca^2+^/CaMK IIβ were simultaneously involved in regulating cell proliferation and apoptosis in human gastric adenocarcinoma cells, BGC-823 cells were treated with those inhibitors, U73122 (PLCγ inhibitor), KN93 (CaMK II inhibitor) and R59949 (DAG-kinase inhibitor), or were transfected with lentiviral-shRNA of PKCδ or CaMK IIβ vectors for different time points, followed by the detection of cell viability using an MTT assay and apoptotic index using DAPI or PI staining. Figure 1A indicated that both CaMK II inhibitor (KN93) and DAG-kinase inhibitor (R59949) led to the decrease of cell viability significantly as well as the effect of PLCγ inhibitor (U73122) (* *p* < 0.05, ** *p* < 0.01, *** *p* < 0.001, **** *p* < 0.0001, *versus* Dimethylsulphoxide (DMSO) group). The cell viability of BGC-823 cells transfected with sh-PKCδ or sh-CaMK IIβ vectors also decreased, compared with sh-Control group (Figure 1B, * *p* < 0.05, ** *p* < 0.01, *** *p* < 0.001, **** *p* < 0.0001). Meanwhile, the apoptotic index (%) increased in BGC-823 cells transfected with sh-PKCδ or sh-CaMK IIβ vectors (Figure 1C,D, * *p* < 0.05, **p < 0.01, *** *p* < 0.001, *versus* sh-Control group). Together, the inhibition of DAG/PKCδ or CaMK IIβ could block cell proliferation or promote cell apoptosis as well as the inhibitory effect of PLCγ1.

The effect of inhibiting DAG/PKCδ and CaMK IIβ on cell migration in human gastric adenocarcinoma cells.

**Figure 2:**
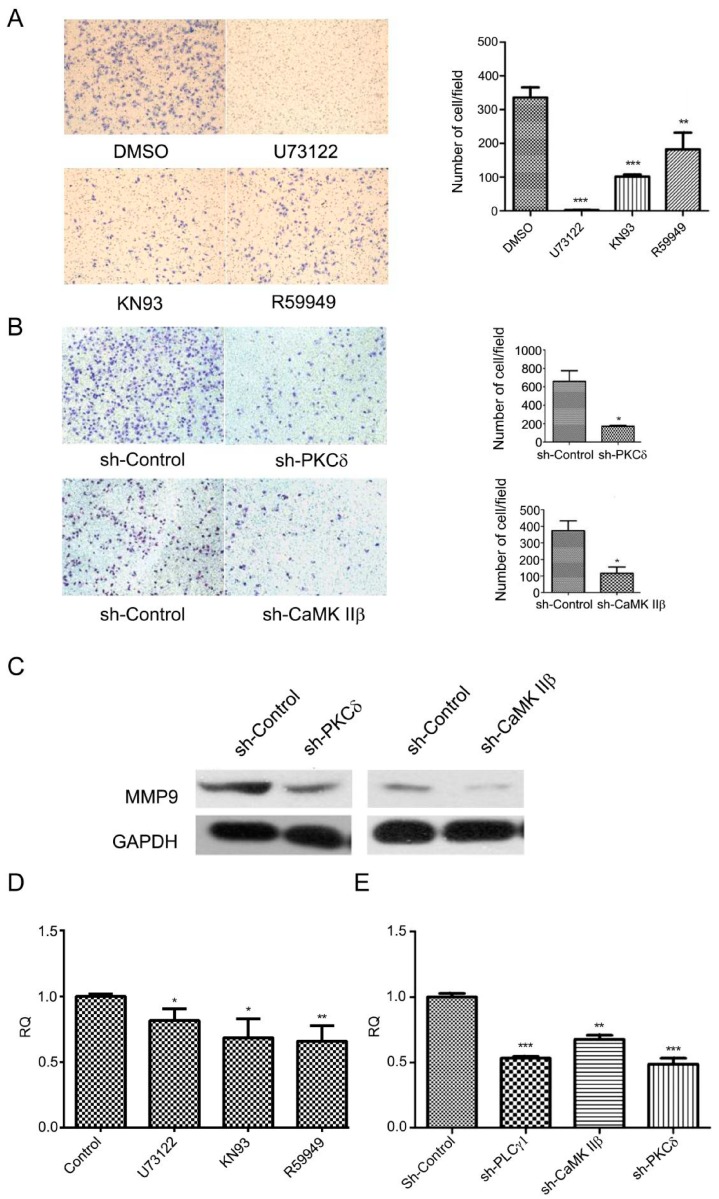
The effect of inhibiting CaMK IIβ and DAG/PKCδ on cell migration in human gastric adenocarcinoma cells. (**A**) Cells were treated with DMSO (2 μL), U73122 (10 μM), KN93 (16 μM), and R59949 (20 μM) for 3 h, respectively. The migration ability were measured by a transwell assay as described in Materials and Methods (magnification 200×); (**B**) Cells were transfected with sh-PKCδ-2 or sh-CaMK IIβ-1 vectors for 48 h, and the migration ability were measured by a transwell assay as described in Materials and Methods (magnification 200×); (**C**) Cells were transfected with sh-PKCδ-2 and sh-CaMK IIβ-1 vectors for 48 h. The expression level of MMP9 was determined by Western blotting analysis as described in Materials and Methods. Glyceraldehyde-3-phosphate dehydrogenase (GAPDH) was used as an internal control; (**D**) Cells were treated with DMSO (2 μL), U73122 (10 μM), KN93 (16 μM), or R59949 (10 μM), respectively. The mRNA levels of MMP9 were measured by RT-PCR analysis as described in Materials and Methods; (**E**) Cells were transfected with sh-PLCγ1, sh-PKCδ-2, or sh-CaMK IIβ-1 vectors for 48 h. The mRNA levels of MMP9 were measured by RT-PCR analysis as described in section Materials and Methods. Data are expressed as mean ± S.D. of three independent experiments, each yielding similar results (* *p* < 0.05, ** *p* < 0.01, *** *p* < 0.001, *versus* control).

Our previous study indicated that the migration of gastric adenocarcinoma cells partly depended on PLCγ1 activation. To investigate the role of IP3/Ca^2+^/CaMK IIβ and DAG/PKCδ axes in cell migration of human gastric adenocarcinoma cells, cells were treated with U73122, KN93, and R59949, respectively, or were transfected with sh-PKCδ or sh-CaMK IIβ vectors, followed the detection of cell migration rate using a Transwell assay and MMP9 expression level with Western blotting analysis. Figure 2A showed that the numbers of migrate cells decreased in response to the treatment by those inhibitors including U73122, KN93, and R59949 (** *p* < 0.01, *** *p* < 0.001, *versus* DMSO group). Similar results were observed in BGC-823 cells with the transfection of sh-PKCδ or sh-CaMK IIβ vectors (Figure 2B, * *p* < 0.05, *versus* sh-Control group). Meanwhile, the expression level of MMP9 protein was down-regulated in BGC-823 cells transfected with sh-PKCδ or sh-CaMK IIβ vectors (Figure 2C). Additionally, the mRNA level of MMP9 was reduced in BGC-823 cells transfected with sh-PKCδ or sh-CaMK IIβ vectors or treated with KN93 or R59949, as well as the effect of sh-PLCγ1 or U73312 (Figure 2D,E, * *p* < 0.05, ** *p* < 0.01, *** *p* < 0.001, *versus* DMSO or sh-Control group). Collectively, the data indicated that the blockade of DAG/PKCδ and CaMK IIβ down-regulated cell migration of gastric adenocarcinoma cells, as well as the effect of PLCγ1 inhibition.

The involvement of Akt, mTOR, S6, and NF-κB signal molecules in regulatory mechanism of IP3/Ca^2+^/CaMK II and DAG/PKCδ axes in human gastric adenocarcinoma cells.

**Figure 3:**
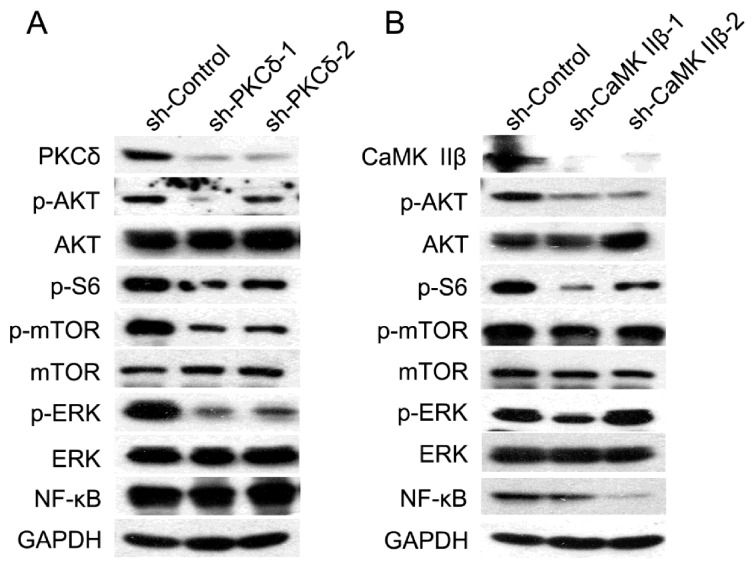
The expression level of Akt, mTOR, S6, and NF-κB in human gastric adenocarcinoma cells with the transfection of sh-PKCδ or sh-CaMK IIβ vectors. (**A**) Cells were transfected with sh-PKCδ-2 vector for 48 h. The expression levels of PKCδ, CaMK IIβ, Akt, p-Akt, mTOR, p-mTOR, p-S6, ERK, p-ERK, and NF-κB were detected by Western blotting analysis as described in Materials and Methods. GAPDH was used as an internal control; (**B**) Cells were transfected with sh-CaMK IIβ vector for 48 h. The expression levels of PKCδ, CaMK IIβ, Akt, p-Akt, mTOR, p-mTOR, p-S6, ERK, p-ERK, and NF-κB were detected by Western blotting analysis as described in Materials and Methods. GAPDH was used as an internal control. Data are representative of three independent experiments, each yielding similar results.

To investigate the regulatory mechanism of the two classical signal axes of PLCγ1, IP3/Ca^2+^/CaMK IIβ and DAG/PKCδ, in cell proliferation and migration of human gastric adenocarcinoma cells, the expression levels of some important signal molecules, including Akt, extracellular signal-regulated kinase (ERK), mTOR, NF-κB, and S6, were detected in BGC-823 cells transfected with sh-PKCδ or sh-CaMK IIβ vectors using Western blotting analysis. The results showed that the transfection of either shRNA-PKCδ or shRNA-CaMK IIβ vectors led to a potent decrease in the phosphorylation level of AKT, mTOR, and S6, without the alteration of total Akt and mTOR (Figure 3A,B). Interestingly, the expression level of NF-κB only was reduced in BGC-823 cells transfected with sh-CaMK IIβ vectors (Figure 3B), whereas, the depletion of PKCδ by shRNA did not affect the expression of NF-κB protein (Figure 3A). Meanwhile, the depletion of PKCδ by shRNA down-regulated the phosphorylation level of ERK, while the transfection with sh-CaMK IIβ vectors did not change the level of p-ERK. As a result, the data indicated that the regulatory mechanism of IP3/Ca^2+^/CaMK IIβ and DAG/PKCδ axes, the two classical axes of PLCγ1, could be associated with the phosphorylation of Akt, mTOR, and S6, while NF-κB or ERK was only involved in the regulation of IP3/Ca^2+^/CaMK II axis or DAG/PKCδ axis, respectively.

The expression of PKCδ and CaMK IIβ in mice tumor xenograft model derived from gastric adenocarcinoma cells with the transfection of sh-PLCγ1.

**Figure 4:**
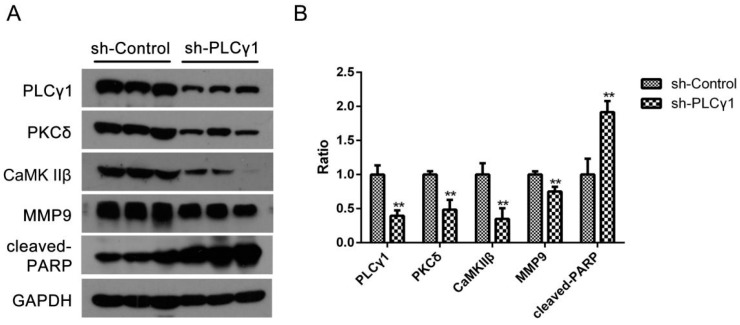
The expression level of PKCδ and CaMK IIβ in mice tumor xenograft model derived from gastric adenocarcinoma cells with the transfection of sh-PLCγ1. Mice were injected human gastric adenocarcinoma cells, BGC-823, for 40 days, and finally sacrified. Tissue protein in subcutaneous tumor tissue was then extracted. (**A**) The expression level of PLCγ1,PKCδ, CaMK IIβ, cleaved-PARP, and MMP9 in the tumor samples were detected by Western blotting analysis as described in Materials and Methods; (**B**) The histogram represents the means ± S.D. of the expression level of PLCγ1,PKCδ, CaMK IIβ, cleaved-PARP, and MMP9 in the tumor samples. (** *p* < 0.01, *versus* control group).

Our other study’s results showed that the depletion of PLCγ1 by shRNA could suppress tumor growth and metastasis in mice tumor xenograft model derived from BGC-823 cells with the transfection of sh-PLCγ1 vector (under review) [19]. Here, we investigated the expression levels of PKCδ and CaMK IIβ protein in the subcutaneous tumor tissue of mice tumor xenograft model using Western blotting analysis. Figure 4 showed that the expression levels of PKCδ and CaMK IIβ protein were down-regulated in tumor tissues as well as the depletion of PLCγ1 by shRNA, while the expression level of MMP9 protein and cleaved-PARP were reduced (** *p* < 0.01). Thus, the data indicated that the levels of PKCδ and CaMK IIβ were down-regulated in the depletion of PLCγ1 by shRNA-mediated tumor growth and metastasis suppression.

## 3. Discussion

In this study, we demonstrated that the inhibition of DAG/PKCδ and CaMK IIβ could block cell proliferation and migration of human gastric adenocarcinoma cells as well as the inhibition of PLCγ1. The expression levels of PKCδ and CaMK IIβ protein were down-regulated in mice tumor xenograft model derived from human gastric adenocarcinoma cells, BGC-823, with the transfection of sh-PLCγ1 vector. Furthermore, both DAG/PKCδ and IP3/Ca^2+^/CaMK IIβ triggered Akt/mTOR/S6 pathway to regulate protein synthesis. Therefore, DAG/PKCδ and IP3/Ca^2+^/CaMK IIβ operate in parallel to each other in PLCγ1-driven cell proliferation and migration of human gastric adenocarcinoma cells.

PLCγ isozymes catalyzes the hydrolysis of PIP2 to generate two intracellular messengers, DAG and IP3, and then triggers DAG/PKC and IP3/Ca^2+^/CaMK II cascade classically. PKCδ, a member of the novel family isoforms, has been a marker of aggressive breast cancers [20,21]. It is described that increased PKCδ in mammary tumor cells relates to transformation and metastatic progression [21]. Furthermore, PKCδ is required for ErbB2 (HER2)-driven mammary gland tumorigenesis and negatively correlates with prognosis in human breast cancer [22]. Increasing concentrations of PKCδ mRNA were associated with reduced overall patient survival (*p* = 0.004) [23]. A very recent study indicates that the pharmacological inhibition of PKCδ impairs LM38-LP cells (derived from a murine mammary papillary adenocarcinoma with tumorigenic and metastatic capacity in BALB/c mice) proliferation through cell cycle arrest [24]. The above studies indicate that PKCδ plays a role in cancer cell proliferation and metastasis. Meanwhile, the involvement of IP3/Ca^2+^/CaMK II axis in cell metabolism has been stated in some studies. IP3/Ca^2+^/CaMK II axis is involved in regulating cholangiocyte growth [25]. CaMK II suppresses the expression of CaMK IV to promote leukemia cell proliferation [26]. TNF-α induced CD44 expression is regulated by AP-1 through the activation of the CaMK-II pathway to mediate cell migration in human monocytic cells [27]. Therefore, both DAG/PKCδ and IP3/Ca^2+^/CaMK II axes play important role in cell proliferation and migration. However, their regulatory effects on cell metabolism are not always identical in the same cell line. In human astroglioma cells, epigallocatechin gallate regulates phosphatidylcholine-specific phospholipase D (PLD) activity via a signaling pathway involving changes in the redox state that stimulates a PLCγ1/IP3/CaMK II/PLD and a PLCγ1/DAG/PKC/PLD axes [28]. The CaMK axis, not the PKC axis, is responsible for the earliest protein phosphorylation event following activation of PLCγ in living *Drosophila* photoreceptors [29,30]. Here, we demonstrated that the inhibitory effect of the depletion of PKCδ or CaMK IIβ by shRNA or the pharmacological inhibiting on cell proliferation and migration was the same as the effect of PLCγ1 inhibition, consistent with other authors’ and our previous studies [9,19,31]. Therefore, it is suggested that the two classical axes of PLCγ1, DAG/PKCδ and IP3/Ca^2+^/CaMK IIβ, are involved simultaneously in PLCγ1-driven cell proliferation and metastasis of human gastric adenocarcinoma cells.

Akt/mTOR pathway is crucial for cell metabolism including cell survival and apoptosis [32], in which mTOR could regulate translation in response to nutrients and growth factors by phosphorylating key components of the protein synthesis machinery, including S6 protein (which is phosphorylated by the S6 kinase) and eIF4G [33]. It is determined that Akt/mTOR/S6 pathway is involved in cell proliferation and apoptosis of cancer cells [34,35]. As an example, the inactivation of Akt/mTOR/S6 pathway is required for an increase in pancreatic cancer cells apoptosis and tumor growth inhibition by thymoquinone pretreatment following gemcitabine treatment synergistically [34].A very recent study also shows that resveratrol synergistically enhanced the antitumor effects of erlotinib in non-small-cell lung cancer cells by inhibiting the Akt/mTOR/S6 pathway [35]. Furthermore, the associations of Akt/mTOR/S6 with PKCδ and CaMK II have been studied. PKCδ is required for ET-1-stimulated or insulin-stimulated mTOR/S6 pathway in adult cardiac muscle cells [36]. The inhibition of PKCδ by rottlerin is supposed to attenuate non-specific airway sensitivity and inflammation through suppressing the PI3K/Akt/mTOR pathway in an asthma model [37]. Meanwhile, our previous study indicated that PLCγ1/IP3/Ca^2+^/CaMK IIβ axis regulated the ECM synthesis of human chondrocytes through triggering mTOR/P70S6K/S6 pathway [38]. Consequently, it is confirmed that the inhibition of PKCδ or CaMK IIβ could suppress the Akt/mTOR/S6 pathway in human gastric adenocarcinoma cells.

Additionally, our data that inhibiting CaMK IIβ led to the down-regulation of NF-κB (one of Akt downstreams) indicate that CaMK IIβ could trigger Akt/NF-κB pathway to resist cell apoptosis, in accordance with previous study that the inhibition of CaMK II prevents PAF-induced priming events including NF-κB activation [39]. Our data is consistent with previous study that DAG/PKC/ERK pathway is involved in the development of glomerular dysfunction in diabetes [40], our finding of the reduced p-ERK by the depletion of PKCδ suggests the existence of DAG/PKCδ/ERK pathway in PLCγ1-driven cell proliferation and migration of human gastric adenocarcinoma cells. The non-alteration of NF-κB or p-ERK level in sh-PKCδ or sh-CaMK IIβ-transduced cells imply that the regulatory effect of PKCδ or CaMK IIβ on NF-κB expression or ERK phosphorylation may be blocked by other signal molecules. We need to further study the complex regulatory mechanism related to PKCδ or CaMK IIβ on NF-κB expression or ERK phosphorylation and validate PKCδ and CaMK IIβ as a molecular biomarker in early diagnosis and disease surveillance.

## 4. Materials and Methods

### 4.1. Reagents and Antibodies

Antibodies against PLCγ1, p-PLCγ1 (Tyr783), PKCδ, CaMK IIβ, Akt, p-Akt (S473), ERK, p-ERK (Thr202/Tyr204), mTOR, p-mTOR (Ser2481), p-S6 (Ser235/236), and NF-κB were purchased from Cell Signaling Technology Inc. (Beverly, MA, USA). Cleaved-PARP, Matrix metalloproteinase-9 (MMP9), and GAPDH were purchased from Abcam (Cambridge, MA, USA). Inhibitors (U73122, KN93, and R59949) were obtained from Sigma-Aldrich in China (Shanghai, China). Other reagents were of the highest grade commercially available.

### 4.2. Cell Culture

The human gastric cancer cell line, BGC-823 cell, was obtained from the Shanghai Institute of Cell Biology, Chinese Academy of Sciences (Shanghai, China), and was maintained in RPMI1640 medium (Thermo Fisher Scienctific, Waltham, MA, USA) supplemented with 10% fetal bovine serum (FBS), 100 U/mL penicillin, and 100 μg/mL streptomycin, at 37 °C in a water-saturated atmosphere of 5% CO_2_.

### 4.3. Plasmid Construction and Transfection

Lentiviral-mediated shorthairpin RNA (shRNA) targeting PLCγ1 (sh-PLCγ1), (5′-CCGGCCTGTGAACCACGAATGGTATCTCGAGATACCATTCGTGGTTCACAGGTTTTTG-3′), sh-PKCδ (Sh1: 5′-CCGGGCAAGACAACAGTGGGACCTACTCGAGTAGGTCCCACTGTTGTCTTGC TTTTTG3′, Sh2: 5′-CCGGGGCCGCTTTGAACTCTACCGTCTCGAGACGGTAGAGTTCAAAGCGGCCTTTTTG-3′), and sh-CaMK IIβ (Sh1: 5′-CCGGGATCATTAAGACCACGGAGCACTCGAGTGCTCCGTGGTCTTAATGATCTTTTTG-3′.Sh2: 5′-CCGGACAAGAAAGCAGATGGAGTCACTCGAGTGACTCCATCTGCTTTCTTGTTTTTTG-3′) were purchased from Gene Chem (Gene Chem, Shanghai, China). The different sh-PLCγ1, sh-PKCδ, and sh-CaMK IIβ vectors were transfected into BGC-823 cells using a lentiviral transfection strategy, respectively. Sh-PLCγ1, sh-PKCδ, and sh-CaMK IIβ stable cell lines were obtained under the pressure of puromycin (2 μg/mL, BioVision, Inc., Milpitas, CA, USA).The expression levels of PLCγ1,PKCδ, and CaMK IIβ protein were detected with Western blotting analysis prior to the other experiments.

### 4.4. MTT Assay

Cells were plated in 96-well plates (1 × 10^4^ cells/well) and cultured for the indicated time. As described as previous studies [41,42], the number of viable cells was detected using 3-(4,5-dimethylthiazol-2-y)-2,5-diphenyl-tetrazolium bromide (MTT) assay.

### 4.5. Apoptosis Assay

Cells were seeded on glass coverslips in 6-well plates for 24 h, rinsed with PBS once, fixed in 4% paraformaldehyde for 10 min, washed three times with PBS, and stained by 50 μg/mL 4,6-diamidino-2-phenylindole (DAPI) stain [43]. The apoptotic cells were observed under Olympus BX41 microscope equipped with a digital camera (Olympus, Tokyo, Japan). Additionally, cells were cultured and stained with Propidium Iodide (PI) stain, followed by flow cytometry (FACScan, Becton Dickinson, Franklin Lakes, NJ, USA) analysis according to previous study [44].

### 4.6. Transwell Assay

According to the previous studies [9,45], 2 × 10^4^ cells in serum-free dulbecco’s modified eagle medium (DMEM) were placed into the upper chambers of Transwell inserts set within wells with 8 µm pore filters, and incubated at 37 °C for 12 h. Cells on the upper surface of the chambers were then removed with cotton swabs. The migrated cells on the lower membrane surface were fixed in methanol and stained with 0.1% Giemsa stain. Eight microscope fields (200×) from each Transwell chamber were randomly selected, and cells adhering to the undersurface of the filter were imaged and counted using an Olympus BX41 microscope equipped with a digital camera (Olympus, Tokyo, Japan).

### 4.7. Western Blot Analysis

Protein extracts were subjected to sodium dodecyl sulfate (SDS)-polyacrylamide gel electrophoresis (PAGE) (8%–10%) and transferred to nitrocellulose membrane for Western blotting analysis [9,42,46]. The membrane was incubated at 4 °C for different time with various antibodies as required, and followed by the addition of the corresponding secondary antibody at room temperature for 1 to 2 h. An enhanced chemiluminescence detection (ECL) kit was used to detect antibody reactivity (Pierce, Rockford, IL, USA).

### 4.8. Real-Time PCR Analysis

Total RNA in cells was extracted using Trizol (Invitrogen, Carlsbad, CA, USA). The amount and quality of the extracted RNA was assessed by spectrophotometry using NanoDrop 2000 (Thermo scientific, Wilmington, DE, USA). cDNA synthesis was performed with 1 µg of total RNA at 37 °C for 15min using the Primescript RT Master Mix Kit (Takara, Dalian, China), and subsequently diluted 10-fold. Real-time PCR was performed using the ABI StepOnePlus Sequence Detection System v2.1 (Applied Biosystems, Foster City, CA, USA) with SYBR Premix Ex Taq II Kit (Takala, Dalian, China). Results were normalized to GAPDH and analyzed using SDS software v2.1 according to previous study [47] .The following primers were used in quantitative PCR for measuring gene expression relative to GAPDH (MMP9: Forward 5′-TGACAGCGACAAGAAGTG-3′, Reverse 5′-CAGTGAAGCGGTACATAGG-3′; GAPDH: Forward 5′-GGAAGGTGAAGGTCGGAGTCA-3′, Reverse 5′-GTCATTGATGGCAACAATATCCACT-3′).

### 4.9. Xenograft Assay in Nude Mice

Thirty-two 6-week-old female BALB/c nu/nu mice were purchased from Shanghai Slac Laboratory Animal Co., Ltd. (Shanghai, China). All animal studies were conducted according to the regulations of the Institutional Animal Care and Use Committee protocol. This study was approved by the Committee on the Ethics of Animal Experiments of the University of Xiamen (ID No. 20110916). Animals bearing tumors were randomly assigned to 4 groups, 200 μL relevant stable cells (sh-Control, sh-PLCγ1, 2 × 10^6^/mouse) in PBS were subcutaneously injected into the right hind leg of mouse, with 8 mice per group [42]. Tumor volume and animal weight were measured every 3–4 days. All animals were killed since injected for 40 days. The protein expression levels of relative signal molecules in these subcutaneous tumors were detected with Western blotting analysis.

### 4.10. Statistical Analysis

The differences between the groups were examined for statistical significance using the Student’s *t*-test with GraphPad Prism 5 software (GraphPad Software, Inc., LaJolla, CA, USA). A value of *p* < 0.05 was considered significant.

## 5. Conclusions

**Scheme 1:**
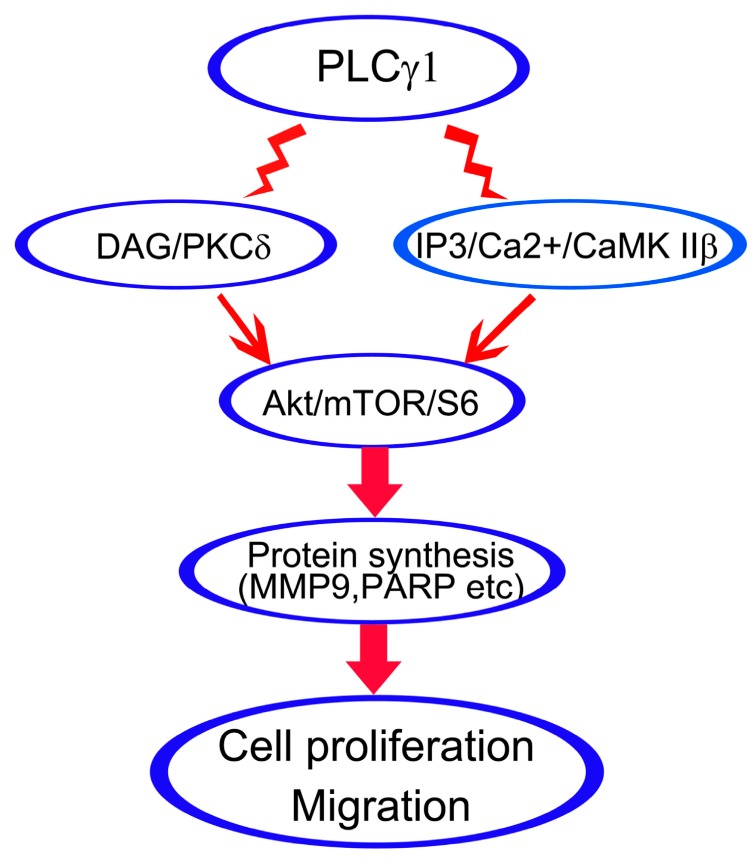
Schematic representation of DAG/PKCδ and IP3/Ca^2+^/CaMK IIβ operate in PLCγ1-driven cell proliferation and migration of human gastric adenocarcinoma cells.

In conclusion, the blockade of both the DAG/PKCδ and IP3/Ca^2+^/CaMK IIβ axes could inhibit cell proliferation and migration, as well as the effect of the inhibition of PLCγ1. Furthermore, their regulatory mechanism is associated with the activation of Akt/mTOR/S6 pathway. Therefore, it is suggested that both DAG/PKCδ and IP3/Ca^2+^/CaMK IIβ operate in parallel to each other in PLCγ1-driven cell proliferation and migration of human gastric adenocarcinoma cells through Akt/mTOR/S6 pathway (Scheme 1).
